# Misinformation surrounding sodium reduction for blood pressure: content analysis of Japanese posts on X

**DOI:** 10.1093/heapro/daae073

**Published:** 2024-06-27

**Authors:** Marina Terada, Tsuyoshi Okuhara, Yuriko Nishiie, Takahiro Kiuchi, Kentaro Murakami

**Affiliations:** Department of Health Communication, Graduate School of Medicine, The University of Tokyo, 7-3-1 Hongo, Bunkyo-ku, Tokyo 113-8655, Japan; Department of Health Communication, School of Public Health, Graduate School of Medicine, The University of Tokyo, 7-3-1 Hongo, Bunkyo-ku, Tokyo 113-8655, Japan; Department of Health Communication, Graduate School of Medicine, The University of Tokyo, 7-3-1 Hongo, Bunkyo-ku, Tokyo 113-8655, Japan; Department of Health Communication, School of Public Health, Graduate School of Medicine, The University of Tokyo, 7-3-1 Hongo, Bunkyo-ku, Tokyo 113-8655, Japan; Department of Social and Preventive Epidemiology, School of Public Health, The University of Tokyo, 7-3-1 Hongo, Bunkyo-ku, Tokyo 113-8655, Japan

**Keywords:** Twitter, X, nutrition, misinformation, sodium, content analysis, blood pressure

## Abstract

This content analysis aimed to assess misinformation themes regarding sodium reduction and blood pressure on X with the goal of providing strategies to address and debunk such misinformation. A total of 531 posts were manually coded into sodium-related misinformation themes, with inclusion criteria for posts asserting no association between sodium reduction and hypertension or claiming consuming sodium is beneficial for health. Numbers and post frequencies per misinformation theme were calculated. Post characteristics, including information sources, advertisements and narratives, were coded, and a correlation analysis was conducted to assess their association with each misinformation theme. Fourteen sodium-related misinformation themes were identified and consistently disseminated on X. The predominant theme, ‘Natural Salt’, accounted for 37.7% (*n* = 200), reaching 1.6 million followers, followed by ‘Reducing salt could be bad for my health’ theme, comprising 28.6% (*n* = 152) and reaching 1.5 million followers. There was a statistical correlation between the natural salt misinformation theme and advertisements. Many of the most frequent misinformation themes identified in this study have not been systematically debunked by organizations such as the World Health Organization and the American Heart Association. This study underscores the importance of continuous monitoring and analysis of sodium-related misinformation on social media platforms and their underlying commercial interests. Such monitoring has the potential to identify prevalent misinformation themes that may pose harm to the public and to inform public health organizations, enabling them to proactively address potential issues through debunking.

**Contribution to Health Promotion**

- Misinformation and myths surrounding sodium reduction have been disseminated through various platforms including social media.
- This content analysis aimed to assess the themes of misinformation regarding sodium reduction on X in Japan.
- Fourteen themes were identified, and the predominant theme was ‘Natural Salt’, accounted for 37.7%, reaching 1.6 million followers.
- WHO and AHA’s debunking efforts may not effectively combat the most frequent misinformation themes as identified in this study.
- Continuous monitoring and analysis of the volume and trends of misinformation themes regarding sodium are imperative for public health organizations.

## INTRODUCTION

A high-sodium intake is a major determinant of hypertension (Mills *et al.*, 2020). Evidence of a causal association between dietary sodium intake and high blood pressure has been reported in observational epidemiological studies and randomized controlled trials (He and Whelton, 2002). A meta-analysis identified an approximately linear relationship between a reduction in sodium intake and a reduction in both systolic and diastolic blood pressure in a dose–response analysis of sodium reduction in clinical trials (Filippini *et al.*, 2021). Based on the entire body of evidence, the World Health Organization (WHO) recommends that all adults aged ≥ 16 years limit their daily salt intake to < 5 g, irrespective of whether they have hypertension (WHO, 2012).

Misinformation and myths surrounding sodium reduction have been disseminated through various platforms. The origin of this misinformation can be traced back to a 1992 study that suggested that lowering salt intake might pose health risks that outweigh the benefits of blood pressure reduction (Muntzel and Drueke, 1992). Notably, this study was financially supported by the salt industry, as pointed out by De Wardener (1999). In 2020 and 2021, a case study by Cappuccio *et al.* (2022) showed that eight studies published in a single journal were propagating the myth that reducing sodium intake does not consistently reduce cardiovascular diseases, but rather that a reduced sodium intake might increase the risk of CVD. These studies did not appropriately declare competing interests and exhibited measurement bias in assessing sodium intake (Campbell *et al.*, 2021; Cappuccio *et al.*, 2022). Cappuccio and colleagues (2014, 2022) demonstrated typical misinformation themes surrounding sodium reduction in response to biased research findings, which are often funded by food companies (Cappuccio and Capewell, 2015). A meta-analysis indicated systematic bias when the research was funded by food and drinks companies (Lesser *et al.*, 2007). These practices of biasing research findings is commonly reported (Moodie *et al.*, 2013), favoring results aligned with the financial interests of industries such as tobacco (Muggli *et al.*, 2003; Holden and Lee, 2009) and alcohol (Bakke and Endal, 2010; Jernigan, 2012). The WHO (2020) and the American Heart Association (AHA) (2022) endeavored to debunk misinformation surrounding sodium.

An increasing number of individuals are turning to social media and online platforms to seek and collect health-related information, including nutrition and hypertension (Saura *et al.*, 2020). However, social media platforms have become conduits for health-related misinformation (Lewis, 2018; Wang *et al.*, 2019) as they can profit financially from the dissemination of misinformation due to increased user engagement (Diaz Ruiz, 2023). This is concerning because the use of social media as an information source is associated with conspiracy beliefs and less health protective measures (Allington *et al.*, 2021), and exposure to health-related misinformation is associated with health behaviors such as being less likely to follow public health guidance and having reduced intention to receive COVID-19 vaccination (Loomba *et al.*, 2021). Regarding hypertension and sodium misinformation, a website analysis conducted on Google in 2021 reported that only 10% of websites, particularly those featured in the first two pages, contained misinformation, suggesting a high level of accuracy in the information provided by these websites (Hussain *et al.*, 2022). In contrast, in 2013, 33% of YouTube videos addressing hypertension were found to contain misleading information (Kumar *et al.*, 2014). In addition, our previous study found that 40.8% of Japanese tweets referring to salt included misinformation, which inserted anti-salt reduction content (Terada *et al*., 2024). The lack of regulation of misinformation on social media platforms, coupled with the promotion of specific products to boost company profits or contradict health evidence by non-qualified individuals or industries of unhealthy food and tobacco, can lead to the spread of misinformation, amplifying public health concerns (Zhou and Zhang, 2007; Zenone *et al.*, 2022). Therefore, efforts to address misinformation must be implemented to minimize these potential harmful effects, although exposure to such misinformation alone do not automatically lead to misperception (Ecker *et al.*, 2022). X is recognized as one of the most common sources of health-related misinformation (Wang *et al.*, 2019; Suarez-Lledo and Alvarez-Galvez, 2021). However, to the best of our knowledge, the specific types of misinformation themes regarding sodium and blood pressure in X have not yet been studied.

In Japan, the prevalence of hypertension was 29.9% and 24.9% in men and women, respectively, who were aged ≥ 20 years in 2019 (Ministry of Health, Labour and Welfare [MHLW], 2019a). The third-term Health Japan 21 initiative by the MHLW has set a target (MHLW, 2023) for the Japanese population to limit salt intake to 7 g/day, which is higher than that set by the WHO (5 g/day). However, the daily salt intake for Japanese men and women was reported to be 10.9 and 9.3 g, respectively, falling short of the established target in 2019 (MHLW, 2019a). Furthermore, 10.8–17.6% of men and 15.4–39.3% of women aged 20–49 years in Japan reported that social media is an information channel that influences eating behaviors (MHLW, 2019a). In addition, 15.3% and 42.8% of the Japanese population reported that they trust or partially trust social media as an information source, respectively (Ministry of Internal Affairs and Communications, Japan, 2021). Given that 40% of the Japanese population utilizes X (Ministry of Internal Affairs and Communications, Japan, 2020) and 38.2% of the Japanese population with hypertension or high blood pressure visits social media (Mitsutake *et al.*, 2023), it is crucial to evaluate the degree to which the population is exposed to misinformation regarding nutrition, particularly sodium, on X.

Therefore, this study aims to assess the volume and themes of misinformation related to sodium reduction and blood pressure in X in Japan. Moreover, numerous studies funded by food companies oppose sodium reduction (Cappuccio *et al.*, 2014), serving as a significant indicator to assess whether misinformation in posts contains advertising or promotional content. Furthermore, misinformation often incorporates advertisement strategies and narratives, making it more appealing than accurate information and resulting in higher levels of user engagement (Garg *et al.*, 2015; Wang *et al.*, 2019). The credibility of information sources has an impact on acceptance of misinformation (Nadarevic *et al.*, 2020; Walter and Tukachinsky, 2020; Ecker *et al.*, 2022). Therefore, insights into advertising, narrative and credibility within misinformation themes are crucial for addressing and comprehending the propagation of misinformation on X. Thus, our research questions are as follows:

RQ1: What are the types of misinformation themes regarding blood pressure and sodium levels in X?

RQ2: Among the most frequent misinformation themes, are there any relationships with the type of information source, linkage with advertisements, or use of narratives?

## METHODS

### Study design

This study carried out a content analysis of retrospective data from X. Terms such as myth, misperception and misleading information have been used interchangeably in various studies. However, in accordance with the definition from previous research, which defines ‘health misinformation as a health-related claim that is based on anecdotal evidence, false, or misleading owing to the lack of existing scientific knowledge’ (Chou *et al.*, 2018; Suarez-Lledo and Alvarez-Galvez, 2021), all these terms are considered as misinformation in this study.

### Data collection

**Fig. 1:**
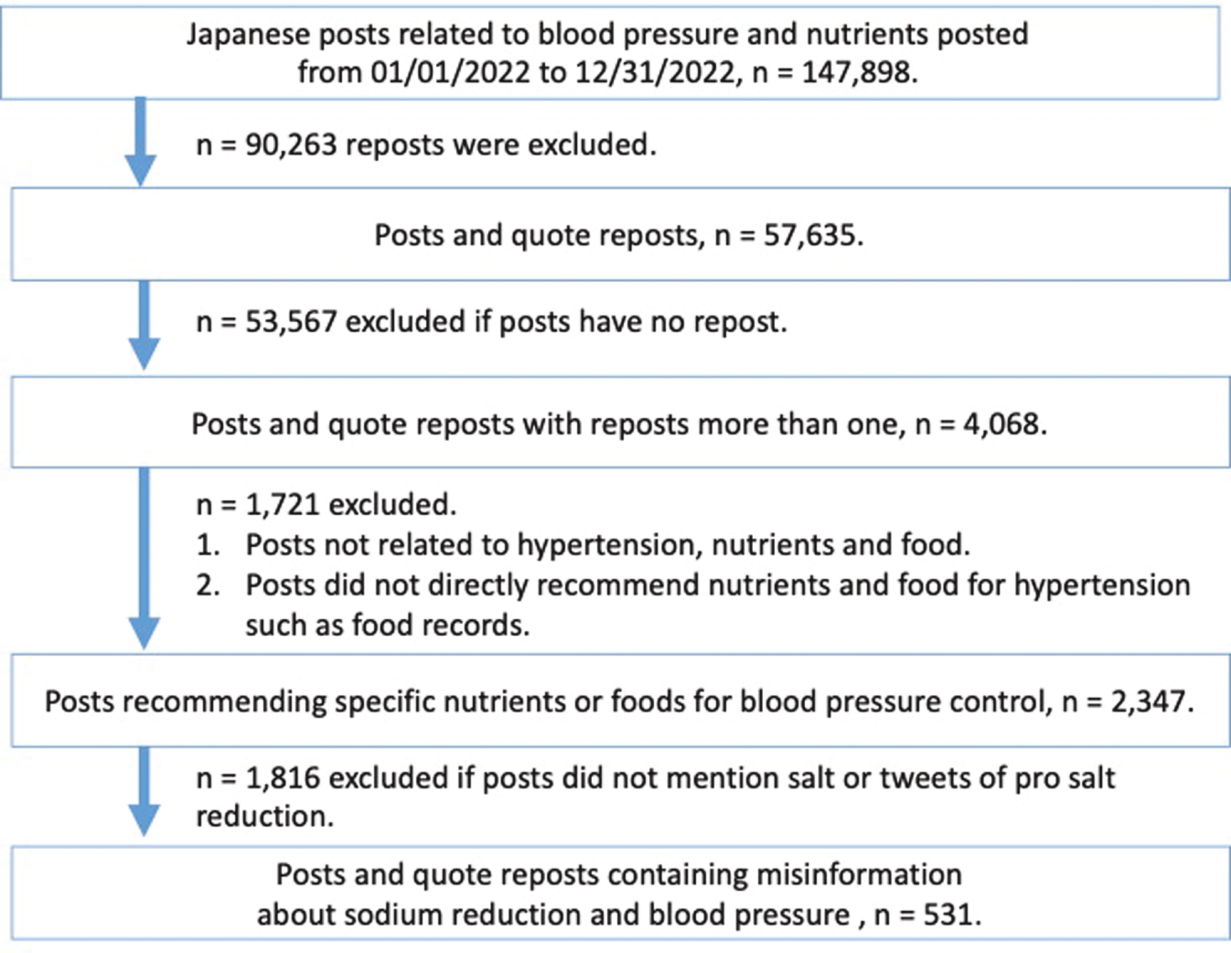
Flow diagram.

We used the Social Insight Service provided by User Local Inc. for data collection (Social Insights, 2024). Social Insight is an aggregate subscription service through which we can purchase data, including posts, likes, reposts and other related information. This study utilized secondary data from our previous research, which analyzed tweets related to nutrients and food recommended for blood pressure control (Terada *et al*., 2024). Between 1 January 2022 and 31 December 2022, we retrospectively studied 147 898 Japanese posts regarding blood pressure, nutrients and food consumption. The keywords were selected based on previous hypertension studies on Facebook, including ‘hypertension’ and ‘blood pressure’ (Al Mamun *et al.*, 2015) and a content analysis of posts related to heart failure and nutrients such as ‘sodium reduction’ and ‘general nutrients’ on X (Hand *et al.*, 2016). Owing to the complexity of the Japanese terms, the keywords used in the search are shown in Supplementary Appendix A. The collected data included content, posting time, usernames, user profile information, number of reposts, likes and follower count. As Figure 1 shows, posts containing keywords related to blood pressure, nutrition and food consumption were collected. After excluding reposts, a total of 57 635 posts remained for analysis. We conducted a descriptive statistical assessment of the number of reposts among the 57 635 posts, finding that the 75th percentile was 0. Consequently, we evaluated posts with more than one repost (beyond the 75th percentile), considering them to be more disseminated. This led to the final selection of 4068 posts for further examination. For these 4068 posts, we conducted manual screening using specific inclusion and exclusion criteria. The inclusion criteria targeted posts recommending the intake of specific foods or nutrients for blood pressure management. Posts unrelated to hypertension or nutrition as well as those not directly mentioning blood pressure (e.g. dietary records) were excluded. Out of the 2347 posts that met the inclusion criteria, we identified those containing misinformation about sodium reduction and blood pressure. These posts either claimed no association between sodium reduction and hypertension or asserted that consuming sodium is beneficial for preventing hypertension, labeling sodium reduction as a conspiracy.

### Misinformation themes

First, this content analysis employed an inductive method to qualitatively code the contents of posts on X. The first author generated preliminary keywords and codes based on consistent patterns that emerged in the process, particularly related to misinformation about sodium reduction and hypertension. Then, these keywords and codes were grouped into themes, forming the foundational categories for misinformation themes used in the subsequent quantitative analysis (Kondracki *et al.*, 2002). During the categorization process, we referenced the misinformation themes proposed by Cappuccio *et al.* (2014, 2022; Cappuccio and Capewell, 2015), a content analysis of YouTube regarding hypertension (Kumar *et al.*, 2014), and the sodium themes debunked by WHO (2020) and AHA (2022). We labeled themes accordingly to arrive at the final misinformation categorization. In cases where a single post contained multiple themes, it was categorized under all applicable themes. For example, if a post stated, ‘Natural salt is healthier than table salt and is good for stabilizing blood pressure’, it would be coded under both the ‘Natural Salt’ and ‘Salt intake is good for blood pressure’ themes. We employed a matrix table to show whether the frequent themes identified in this study had been debunked by WHO (2020) and AHA (2022), as well as themes from previous studies that analyzed misinformation surrounding sodium reduction and blood pressure (Cappuccio *et al.*, 2014; Kumar *et al.*, 2014; Cappuccio and Capewell, 2015, 2022).

### Posts’ characteristics: sources of information, advertisement and narrative

We coded the posts to determine whether the misinformation themes included information sources, advertisements and narratives. Following the definition of a previous study (Hinyard and Kreuter, 2007), we coded narratives if posts mentioned ‘official stories’, ‘invented stories’, ‘firsthand experiential stories’, ‘secondhand stories’ and ‘culturally common stories’. Meanwhile, if the content of the posts promoted specific brands or products for blood pressure control, we coded them as advertisements. Regarding information sources, we adopted a definition from a previous content analysis of Japanese websites related to nutrition (Murakami *et al.*, 2023). If posts included peer-reviewed articles, nonfiction books, Dietary Reference Intake (DRI) (MHLW, 2019b), Japanese dish-based dietary guidelines (e.g. Food Guide Spinning Top) (Ministry of Health, Labour and Welfare and the Ministry of Agriculture, 2005) and other information sources published by a public organization, we considered them to have appropriate information sources. However, posts that referred to a source such as ‘according to a study’ but without providing a specific citation, or mentioning news articles without explicit citations, were categorized as having information sources without appropriate citations.

### Coding procedure

The first author, M.T., coded all the data from the initial 2347 posts and developed a coding manual. Subsequently, the first (M.T.) and third authors (Y.N.) underwent joint training to code the first 30 posts using the coding manual. Microsoft Excel (ver. 16.70) was employed for coding. Following the training, the third author coded 20% of the total eligible data, and inter-rater reliability was calculated for all categories.

### Statistical analysis

**Fig. 2:**
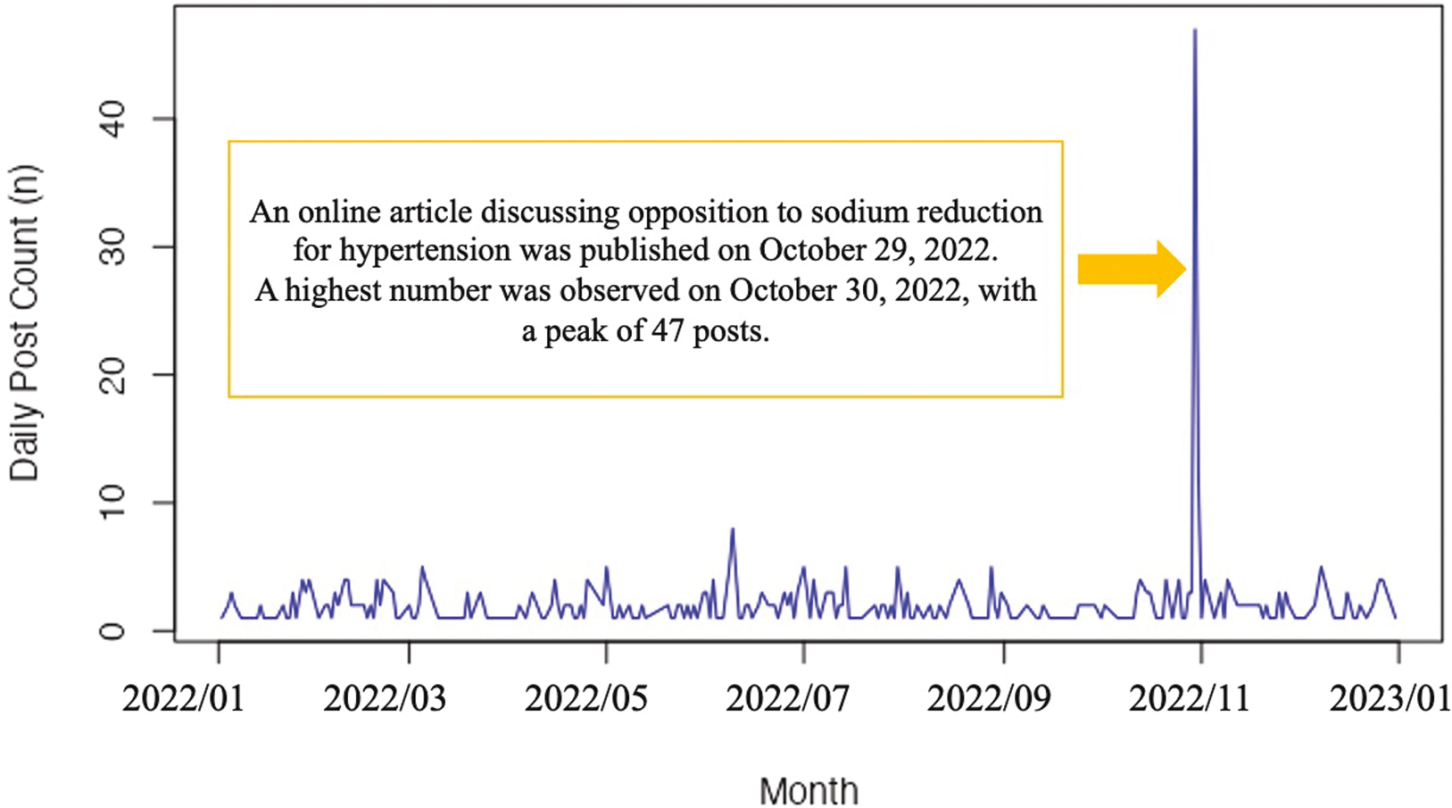
Daily post count related to sodium-related misinformation from 1 January 2022 to 31 December 2022.

Figure 2 illustrates the daily post count, providing insights into the trend of posts from 1 January 2022 to 31 December 2022. To examine the volume and impact of misinformation themes (RQ1), descriptive analysis was conducted to calculate the number and proportion of themes in the posts. In addition, the number of reposts and followers for each theme were calculated. The sum of followers for users of each post was calculated to illustrate the potential exposure within each misinformation theme. To assess the statistical correlations between each misinformation theme and the characteristics of the information sources, advertisements, and narratives, Cramer’s V coefficients were calculated, in addition to the chi-square test (Riffe *et al.*, 2005). Two-tailed chi-square tests were used to evaluate the significance of the relationship between various categorical data, and the significance level was set at α = 0.05. Correlation analysis using Cramer’s V demonstrated the strength of these relationships. Cramer’s V is a statistical measure that ranges from 0 to 1, where 0 indicates no association and 1 indicates perfect association. Due to the low percentages of each category, this study utilized the Gwet AC1 statistic, considering its robustness compared to Cohen’s kappa coefficient (Gwet, 2008; Nishiura, 2010; Wongpakaran *et al.*, 2013). The Gwet AC1 statistic was calculated for each misinformation theme and the characteristics of the information sources, advertisements and narratives. Statistical analyses were performed using R for MacOS.

### Ethical consideration

This study was approved by the Ethics Committee of the Graduate School of Medicine at the University of Tokyo (2022288NI). The data underlying this article were provided by User Local Inc. for data collection (Social Insights, 2024) under purchase. Data will be shared on request to the corresponding author with permission of User Local Inc.

## RESULTS

### Description of the sample and data trends

The final sample included 531 posts, each with more than one repost, containing misinformation about sodium reduction and blood pressure. The number of unique users was 364. Among the 531 posts, 6.2% (*n* = 33) were posted by a single user, making them the most frequent user. As depicted in Figure 2, posts related to anti-sodium reduction were consistently shared throughout the year. Notably, the number of posts increased on 30 and 31 October, reaching 47 posts and 12 posts, respectively. The posts on 30 October, the day with the highest number of posts (95.7%, *n* = 45/47), as well as the posts on 31 October (100%, *n* = 12/12) cited an online news article. The article, published on 29 October, was titled ‘The Health Benefits of Salt Reduction Are a Lie, because Longevity is Achieved by Consuming High Salt in the Japanese Population’.

### Misinformation themes related to reduction in sodium intake

Supplementary Appendix B presents the misinformation themes, post characteristics, keywords and examples related to blood pressure and sodium reduction. Fourteen misinformation themes were identified. Table 1 shows the definitions for each theme, along with the number (*n*), percentage (%), quartiles of the number of reposts for each theme and the total number of followers. The Gwet AC1 statistic for each theme and the post characteristics ranged from 0.76 to 1.00. The seven most frequent themes, exposed to more than 100 000 followers, were ‘Natural Salt’, ‘Reducing salt could be bad for my health’, ‘Salt intake is good for blood pressure’, ‘Conspiracy theory’, ‘Negation of blood pressure medication’, ‘Japanese populations with higher salt intake have longer lifespans’ and ‘Misconception of blood pressure standards’. Among them, the most frequent theme was ‘Natural Salt’, accounted for 37.7% (*n* = 200) and reaching 1 594 182 followers. The second most frequent theme was ‘Reducing salt could be bad for my health’, accounted for 28.6% (*n* = 152) and reaching 1 456 590 followers. Due to the themes asserting that salt reduction is bad for health, such as the claim that sodium reduction increases the risk of dementia, a detailed classification was conducted, and the results are presented in Supplementary Appendix C. Seventeen specific diseases or health concerns were identified within this theme, while others generally stated that a reduction in sodium intake is harmful to health but without specifying particular conditions. The majority of posts in this theme mentioned that sodium reduction increases the risk of diseases such as dementia (*n* = 62, 40.8%) and cerebral infarction (*n* = 51, 33.6%). The third most frequent theme was ‘Salt intake is good for blood pressure’, accounted for 28.1% (*n* = 149) and reaching 1 408 653 followers. This was followed by the ‘Conspiracy theory’ theme, accounted for 20.0% (*n* = 106). Furthermore, in conjunction with the misinformation related sodium reduction, ‘Negation of blood pressure medication’ was the fifth most frequent theme, accounting for 19.8% (*n* = 105) of posts. ‘Japanese populations with higher salt intake have longer lifespans’ ranked sixth, accounted for 12.1% (*n* = 64), while ‘Misconception of blood pressure standards’ was the seventh most frequent theme, accounting for 6.6% (*n* = 35) of posts.

**Table 1:** Themes of misinformation regarding sodium reduction and blood pressure, and descriptive statistics of reposts, posted in 2022

Themes of misinformation	Definition	*n*	%	Reposts IQR	Sum of followers
Natural salt	Natural salt is healthy and manufactured salt is bad for health.	200	37.7%	3.0 (1.0–13.3)	1,594,182
Reducing salt could be bad for my health/Salt intake is good for health	Salt reduction is bad for health, and salt intake is actually good for health.	152	28.6%	2.0 (1.0–25.5)	1,456,590
Salt intake is good for blood pressure	Salt intake is good for blood pressure.	149	28.1%	4.0 (1.0–15.0)	1,408,653
Conspiracy theory	The reduction of salt intake being motivated by profit-seeking from government or pharmaceutical companies.	106	20.0%	5.5 (2.0–33.8)	792,855
Negation of blood pressure medication	Denying the effectiveness of antihypertensive medications.	105	19.8%	2.0 (1.0–5.0)	736,916
Japanese populations with higher salt intake have longer lifespans	Japanese populations with higher salt intake have longer lifespans, implying that reducing salt intake may not be meaningful for health.	64	12.1%	2.0 (1.0–5.0)	230,730
Misconception of blood pressure standards	The established blood pressure standards are incorrect. A higher standard such as 180 mmHg is considered acceptable.	35	6.6%	2.0 (1.0–7.5)	125,417
Hot climates or when we exercise	We need sodium in hot climates or when we exercise because we sweat a lot.	18	3.4%	3.5 (2.0–23.8)	58,702
Salt sensitivity	Only individuals with salt sensitivity need to be cautious about their salt intake.	12	2.3%	2.0 (1.0–3.0)	37,713
Lack of evidence	The evidence for the health benefits of salt reduction has not been established.	12	2.3%	2.0 (1.0–4.0)	44,609
Reducing salt intake leads to sodium deficiency	Reducing salt intake can lead to a deficiency of essential sodium needed for bodily functions.	9	1.7%	15.0 (2.0–25.0)	102,551
Our body needs sodium	Our body needs sodium, thereby advocating for increased salt consumption.	6	1.1%	2.0 (1.0–5.3)	9,295
No causal relationship with stroke or myocardial infarction	Salt reduction has no effect on preventing stroke or heart attack.	4	0.7%	2.5 (2.0–4.0)	18,738
Misconception of salt intake standards	The recommended limit of 6 g for salt intake is incorrect. A higher amount is acceptable.	2	0.4%	6.5 (3.8–9.3)	1,512
Characteristics of Posts					
Information sources with citations	Peer-reviewed articles, nonfiction books, dietary reference intake, Japanese dish-based dietary guidelines and other references published by a public organization.	3	0.6%	1.0 (1.0–14.0)	2,808
Information sources without appropriate citations	News articles, reference without citation such as peer-reviewed articles or books.	125	23.5%	1.0 (1.0–3.0)	792,674
Narrative	Posts contain narratives.	107	20.2%	4.0 (1.0–13.0)	868,347
Advertisement	Promotion of specific products or goods for blood pressure control.	32	6.0%	4.0 (2.0–8.3)	281,738

One post can contain multiple themes, leading to a cumulative number of posts that may surpass the total count of 531. The denominator for the percentage is the total number of posts, which is 531. It represents the proportion of each theme in the final sample of posts. The sum of followers for users of each post was calculated to illustrate the potential exposure within each misinformation theme. IQR, interquartile range.

Table 2 shows whether the 14 most frequent misinformation themes on Japanese posts on X identified in this study were addressed in the debunking efforts by WHO, AHA and previous research (Cappuccio *et al.*, 2014, 2022; Kumar *et al.*, 2014; Cappuccio and Capewell, 2015). The exception of the top two themes, most of the frequent themes identified in this study were not debunked by them. The matrix of the remaining less frequent themes in this study and the debunking efforts are presented in Supplementary Appendix D.

**Table 2:** Matrix of misinformation themes presented by WHO, AHA, previous studies and this study, ordered by high proportion of posts in this study

Themes	Our study	AHA	WHO	Cappuccio *et al*. (2022)	Cappuccio and Capewell (2015)	Cappuccio *et al*. (2014)	Kumar *et al*. (2014)
Sea salt is not ‘better’ than manufactured salt simply because it is ‘natural’/Sea salt has less sodium than table salt/Rock salt, sea salt or other expensive salts are more healthful than table salt	レ	レ	レ	レ	レ		
Reducing salt could be bad for my health/Sodium intake below 3.0 g per day (7.5 g of salt per day) could be potentially harmful	レ		レ	レ	レ	レ	
Salt intake is good for blood pressure	レ						
Conspiracy theory	レ						
Misinformation regarding effects of pharmacologic treatment	レ						レ
Japanese populations with higher salt intake have longer lifespans	レ						
Misconception of blood pressure standards	レ						
On a hot and humid day when you sweat, you need more salt in the diet/We need sodium in hot climates or when we exercise because we sweat a lot	レ		レ	レ	レ		
Salt sensitivity	レ						
Lack of evidence	レ						
Reducing salt intake leads to sodium deficiency	レ						
Our body needs sodium	レ			レ	レ		
No causal relationship with stroke or myocardial infarction/Minimized health risks associated with HTN	レ						レ
Misconception of salt intake standards/The current sodium intake is a physiologically set normal range in adult humans/The ‘normal’ sodium intake is between 5.0 and 7.5 g per day (12.5 and 18.8 g salt per day) and a ‘moderate’ intake between 3.0 and 5.0 g per day (7.5 and 12.5 g salt per day)	レ			レ	レ	レ	

This table only includes misinformation themes that this study identified. Other themes presented by other sources are shown in Supplementary Appendix D. HTN, hypertension.

### Relationship between prevalent misinformation and sources of information, advertisements and narratives

**Fig. 3:**
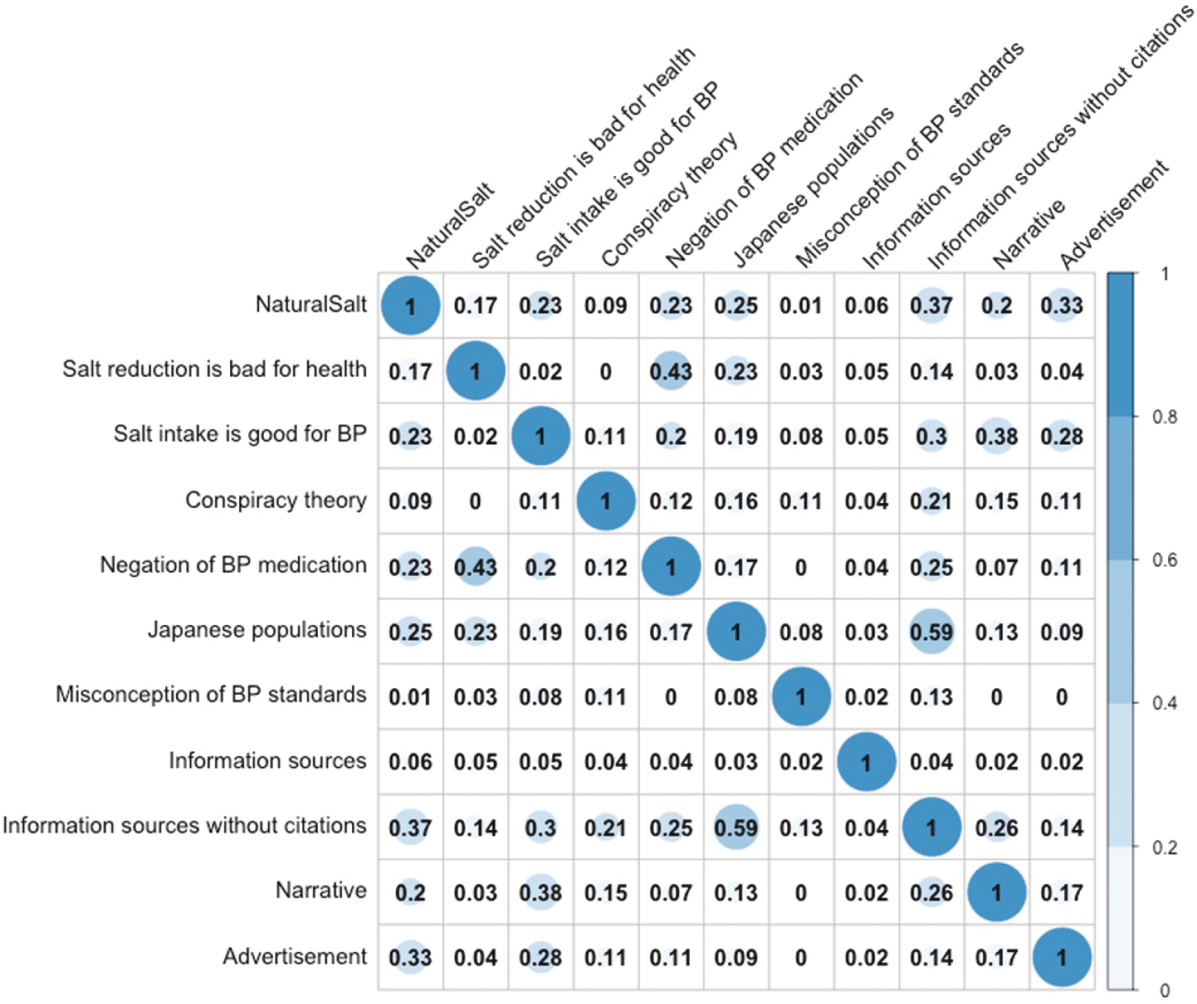
Correlation matrix illustrating the strength of relationships between misinformation themes and post characteristics linked to information sources, narratives, and advertisements, using Cramer’s V coefficient. BP, blood pressure.

Figure 3 shows the strength of the relationships between misinformation themes and post characteristics related to information sources, narratives and advertisements, as measured by Cramer’s V coefficient. The highest correlation (0.59) was observed between the theme ‘Japanese populations with higher salt intake have longer lifespans’ and information sources lacking appropriate citations. The second-highest Cramer’s V coefficient (0.43) was observed between ‘Reducing salt could be bad for my health’ and ‘Negation of blood pressure medication’. The ‘Salt intake is good for blood pressure’ theme showed Cramer’s V coefficients of 0.38 and 0.3 in relation to narrative and information sources without appropriate citations, respectively. The ‘Natural Salt’ theme showed a Cramer’s V coefficient of 0.33 in relation to advertisements. In Supplementary Appendix E, results with a chi-square test *p*-value of < 0.05 and a Cramer’s V coefficient of ≥ 0.3, indicating a significant correlation, are highlighted.

## DISCUSSION AND CONCLUSION

### Discussion

This content analysis identified a total of 531 posts, with more than one repost (above the 75th percentile), spreading misinformation regarding sodium and blood pressure throughout 2022. Following the dissemination of online news critical of a reduction in sodium intake on 29 October 2022, there was a highest number in posts containing misinformation about sodium reduction within the subsequent 1–2 days. Most of these posts quoted the news article, indicating that the spread of misinformation on this topic on X was triggered by the news article. To our knowledge, this is the first study to examine the types of misinformation themes surrounding sodium reduction and blood pressure on social media, specifically on X.

The most frequent theme in this study is ‘Natural Salt’, which has been debunked by previous research (Cappuccio and Capewell, 2015; Cappuccio *et al.*, 2022), WHO (2020) and AHA (2022), marking it as a frequently disseminated piece of misinformation regarding sodium, not only in Japan but also globally. With only 200 posts, the theme reached over 1.6 million followers, potentially influencing public health beliefs regarding eating behaviors. One example of this theme was the claim that ‘Natural salt is healthier than manufactured salt because it contains less sodium and more minerals, including potassium, so you can consume as much as you want’, and with the specific name of the brand of salt being mentioned. However, research indicates that most Japanese and imported natural salts, including the brands mentioned in the study, contain ~ 97% sodium, excluding water (Niino *et al*., 2003; Sasaki, 2023). These natural salts reportedly contain ~ 1% potassium in the case of Japanese varieties and 0.2–1% potassium in imported varieties, respectively (Niino *et al*., 2003). Moreover, manufactured salt has been shown to contain ~ 99.6% sodium, as reported by the Standard Tables of Food Composition in Japan (Ministry of Education, Culture, Sports, Science and Technology, 2020). Prior studies (Cappuccio *et al.*, 2022), as well as WHO (2020) and AHA (2024), have not supported the claim that natural salt is healthier than manufactured salt, and there is no established evidence to back such assertions. In addition, there was a statistical correlation between the natural salt theme and advertisements. Considering the concerns raised about the potential influence of food companies in scientific papers with a negative stance on sodium reduction (Cappuccio *et al.*, 2014; Cappuccio and Capewell, 2015) and considering the correlation with advertisements in the posts, it may be reasonable to consider the possibility that the food industry is involved in the dissemination of misinformation on sodium reduction on X. This assertion aligns with previous studies which found that half of misleading YouTube videos related to hypertension included advertisement (Kumar *et al.*, 2014). In addition, a small subset of anti-vaccine advertisers has utilized Facebook advertisements to target specific audiences to spread anti-vaccine content (Jamison *et al.*, 2020). This suggests the possibility of social media playing a role in spreading health misinformation to benefit certain salt-related industries or individuals by promoting products and contradicting established evidence that goes against their profit motives (Zenone *et al.*, 2022). This is concerning as exposure to industry-sponsored messages, such as those by tobacco and sugary beverage companies, led to greater uncertainty or false certainty regarding the risks of their products on health compared to exposure to non-industry messages (Maani *et al.*, 2022).

The second most frequent misinformation theme was ‘Reducing salt could be bad for my health’, which aligns with the theme debunked by WHO and Cappuccio *et al.* (2014, 2022; Cappuccio and Capewell, 2015), and reached 1.45 million followers across 152 posts. Most posts under this theme mentioned that sodium reduction results in an increased risk of dementia and cerebral infarction. However, a high-sodium diet has been identified as an independent risk factor for dementia and stroke (Fiocco *et al.*, 2012; Gardener *et al.*, 2012; Blumenthal *et al.*, 2019; Kendig and Morris, 2019). Furthermore, according to the 2019 Global Burden of Disease data in Japan, a high-sodium diet was ranked eighth in all-ages disability-adjusted life years (DALYs) attributable to preventable behavioral risk factors and ninth in all-age number of deaths attributable to preventable behavioral risk factors (Nomura *et al.*, 2022). This highlights that sodium intake is a significant dietary risk behavior contributing to DALYs and deaths. However, misinformation contradicting these research findings has been frequently disseminated on X. Furthermore, it was observed that this theme is associated with the ‘Negation of blood pressure medication’ theme. This implies that efforts to debunk misinformation about sodium should encompass the dissemination of accurate information regarding blood pressure medications as well.

The misinformation themes ‘Salt intake is good for blood pressure’, ‘Conspiracy theory’ and ‘Negation of blood pressure medication’ were among the most frequent; however, none of them had been debunked by WHO and AHA. The ‘Salt intake is good for blood pressure’ theme was correlated with information sources without appropriate citations; for example, by stating ‘A study reports that increased sodium intake is good for blood pressure’ in the post but without providing specific details of the research. Effectively debunking these misinformation themes, by undermining the plausibility of the misinformation or the credibility of its source, could be crucial in changing recipients’ beliefs (Ecker *et al.*, 2022). Furthermore, this theme is linked to narratives. For instance, some posts provided personal examples such as, ‘I add one scoop of (specific salt brand) into my bottled water daily, and my blood pressure has remained stable’. Such narratives could be harmful and particularly appealing to followers, as previous studies have shown that narrative stories attract more viewership on social media, including YouTube (Kumar *et al.*, 2014; Garg *et al.*, 2015). Regarding the ‘Negation of blood pressure medication’ theme, a content analysis of YouTube also identified the dissemination of this theme in relation to blood pressure (Kumar *et al.*, 2014). Moreover, considering the concern that only ~ 50% of the Japanese population with hypertension receives pharmacological treatment (Hirawa *et al.*, 2019; Zhou *et al.*, 2019, 2021), it is essential to combat and debunk the misinformation negating the effectiveness of blood pressure medications.

The misinformation theme suggesting that ‘Japanese populations with higher salt intake have longer lifespans’ might be unique within the Japanese context on X. Sodium intake is a major determinant of blood pressure elevation (Mills *et al.*, 2020) and contributes to mortality and DALYs in the Japanese population (Nomura *et al.*, 2022). The theory that sodium intake does not contribute to mortality or DALYs due to Japan’s longer average lifespan compared with other countries has not been substantiated. Furthermore, this theme correlated with information sources without an appropriate citations of news articles, indicating that it was prompted by online news and therefore also became a more disseminated theme. In Japan, measures against misinformation related to sodium reduction appear to be insufficient, especially by organizations such as the MHLW and the Japanese Society of Hypertension (JSH). The JSH has taken steps, such as the establishment of the JSH Tokyo declaration, to advocate for sodium reduction as well as proposed educational activities related to sodium for citizens (Tsuchihashi, 2022). Yet, this study suggests the need for additional efforts in disseminating accurate information about sodium reduction to the public and implementing strategies to counter misinformation, such as debunking, beyond the current activities of the MHLW and the JSH.

The implications of this study are as follows: firstly, we advocate for continuous monitoring of sodium-related misinformation themes and their exposure on a global scale, along with an assessment of their actual impact on people’s health behaviors and their underlying commercial interests. This aligns with a research priority, stated by The World Hypertension League, Resolve to Save Lives and the International Society of Hypertension (Campbell *et al.*, 2022). As the most frequent misinformation themes identified in this study were not comprehensively covered by WHO and AHA, efforts to correct misinformation should prioritize themes that are widely exposed to the public and pose a risk to individuals’ health (Lewandowsky *et al.*, 2020; Ecker *et al.*, 2022). Next, social media platforms themselves should take approaches to regulate misinformation and exercise caution with harmful advertisements by industries that contradict evidence, as observed in this study. Health-harming industries utilize social media to promote their products to defined groups (Zenone *et al.*, 2022), and certain populations such as those who have lower knowledge and literacy are susceptibility to health misinformation (Nan *et al.*, 2022). Intervention and debunking efforts should be targeted towards populations at risk. However, current algorithms and business decisions on social media platforms may contribute to the spread of health misinformation (Zenone *et al.*, 2022; Gilmore *et al.*, 2023). The business model of social media platforms aims to maximize consumer engagement, which encompasses the time and attention users spend on the platform (Diaz Ruiz, 2023). Within this ecosystem, there is often an incentive for users to create content that has the potential to go viral, leading to financial rewards for circulating controversial claims, adversarial narratives and misinformation, rather than minimizing harm to the public (Braun and Eklund, 2019; Diaz Ruiz, 2023). Individuals might encounter misinformation more frequently if they have previously been exposed to sodium-related misinformation. Algorithms might prioritize content based on advertising purposes or users’ past interactions, including within echo chambers. This exposure can reinforce their misperceptions due to confirmation bias (Cinelli *et al.*, 2021). There is a need to restructure the platforms’ infrastructures to remove financial incentives for spreading misinformation (Diaz Ruiz, 2023). Another possible solution to address this issue is for health organizations and social media platforms to consider expanding collaboration to regulate misinformation across a broader range of topics, including those related to sodium. This recommendation is supported by the successful collaboration between the WHO and corporations such as YouTube and Meta to regulate COVID-19 misinformation, resulting in the removal of a half of million misinformation videos on YouTube (Wojcicki, 2021) and the provision of accurate and trustworthy information to the general public (Germani *et al.*, 2022). Lastly, we advocate for experts, scientists, governments, organizations and other stakeholders to encourage this momentum in combating circulated sodium-related misinformation and promoting the reduction of sodium intake at a population level. As noted in a global statement by Campbell *et al.* (2022), scientific organizations are now working together to support achievement of the WHO-recommended sodium intake levels in each nation and globally. Building scientific evidence, policy development, advocacy and different actions and perspectives toward sodium reduction are needed.

This study has several limitations. First, it solely focused on capturing misinformation themes present in Japanese posts on X. Consequently, the identified misinformation in this study may be heavily influenced by the Japanese context. However, it is noteworthy that the first and second most frequent themes had already been debunked by global organizations and prior research in another country, indicating their universal nature. Second, due to the nature of the data, the assessment of exposure relied on the follower count of users who propagated misinformation. It is important to acknowledge the potential existence of echo chambers on social media, where duplicated users may have been included. Future research could explore collecting follower IDs to assess exposure based on the number of unique users. Third, the final sample was restricted to tweets with more than one repost. This approach may overlook posts with zero reposts but with a significant number of likes and followers. Fourth, this study exclusively extracted posts from 2022, observing trends in posts and misinformation themes coinciding with the release of online news. It is important to note that the observed trend and misinformation themes may vary over a longer period. Despite these limitations, the study contributes to the literature by classifying misinformation themes related to sodium reduction and blood pressure on platform X.

### Conclusion

This content analysis indicates the dissemination of misinformation related to sodium reduction and hypertension on platform X. The involvement of individuals and the food industry in disseminating misinformation on sodium reduction on X for profit motives is a possibility, given the statistical relationship observed between advertisements. Despite the ongoing debunking efforts by WHO and AHA to combat misinformation, there is a concern that they may not systematically address the most frequent misinformation themes surrounding sodium reduction and blood pressure, as identified in this study. To effectively address misinformation, continuous monitoring and analysis of the volume and trends of misinformation themes regarding sodium are imperative for public health organizations, not only in Japan but also globally.

## SUPPLEMENTARY MATERIAL

Supplementary material is available at *Health Promotion International* online.

daae073_suppl_Supplementary_Material

## References

[CIT0001] Al Mamun, M., Ibrahim, H. M. and Turin, T. C. (2015) Social media in communicating health information: an analysis of Facebook groups related to hypertension. Preventing Chronic Disease, 12, 140265. 10.5888/pcd12.140265 PMC4310711 25633486

[CIT0002] Allington, D., Duffy, B., Wessely, S., Dhavan, N. and Rubin, J. (2021) Health-protective behaviour, social media usage and conspiracy belief during the COVID-19 public health emergency. Psychological Medicine, 51, 1763–1769. 32513320 10.1017/S003329172000224X PMC7298098

[CIT0003] American Heart Association (AHA) (2022) 7 Salty Sodium Myths Busted Infographic. https://www.heart.org/en/healthy-living/healthy-eating/eat-smart/sodium/7-salty-sodium-myths-busted-infographic (last accessed 27 June 2023).

[CIT0004] American Heart Association (AHA) (2024) Sea Salt vs. Table Salt. https://www.heart.org/en/healthy-living/healthy-eating/eat-smart/sodium/sea-salt-vs-table-salt (last accessed 2 May 2024).

[CIT0005] Bakke, O. and Endal, D. (2010) Vested interests in addiction research and policy alcohol policies out of context: drinks industry supplanting government role in alcohol policies in sub‐Saharan Africa. Addiction, 105, 22–28. 20078460 10.1111/j.1360-0443.2009.02695.x PMC2805868

[CIT0006] Blumenthal, J. A., Smith, P. J., Mabe, S., Hinderliter, A., Lin, P.-H., Liao, L. et al. (2019) Lifestyle and neurocognition in older adults with cognitive impairments: a randomized trial. Neurology, 92, e212–e223. 30568005 10.1212/WNL.0000000000006784 PMC6340382

[CIT0007] Braun, J. A. and Eklund, J. L. (2019) Fake news, real money: ad tech platforms, profit-driven hoaxes, and the business of journalism. Digital Journalism, 7, 1–21.

[CIT0008] Campbell, N. R. C., He, F. J., Cappuccio, F. P. and MacGregor, G. A. (2021) Dietary sodium ‘controversy’—issues and potential solutions. Current Nutrition Reports, 10, 188–199. 34146234 10.1007/s13668-021-00357-1

[CIT0009] Campbell, N. R. C., Whelton, P. K., Orias, M., Wainford, R. D., Cappuccio, F. P., Ide, N. et al. (2022) 2022 World Hypertension League, resolve to save lives and international society of hypertension dietary sodium (salt) global call to action. Journal of Human Hypertension, 37, 428–437. 35581323 10.1038/s41371-022-00690-0 PMC9110933

[CIT0010] Cappuccio, F. P., Campbell, N. R. C., He, F. J., Jacobson, M. F., MacGregor, G. A., Antman, E. et al. (2022) Sodium and health: old myths and a controversy based on denial. Current Nutrition Reports, 11, 172–184. 35165869 10.1007/s13668-021-00383-z PMC9174123

[CIT0011] Cappuccio, F. P. and Capewell, S. (2015) Facts, issues, and controversies in salt reduction for the prevention of cardiovascular disease. Functional Food Reviews, 7, 41–61.

[CIT0012] Cappuccio, F. P., Capewell, S., He, F. J. and MacGregor, G. A. (2014) Salt: the dying echoes of the food industry. American Journal of Hypertension, 27, 279–281. 24356574 10.1093/ajh/hpt216

[CIT0013] Chou, W.-Y. S., Oh, A. and Klein, W. M. P. (2018) Addressing health-related misinformation on social media. JAMA, 320, 2417. 30428002 10.1001/jama.2018.16865

[CIT0014] Cinelli, M., De Francisci Morales, G., Galeazzi, A., Quattrociocchi, W. and Starnini, M. (2021) The echo chamber effect on social media. Proceedings of the National Academy of Sciences of the United States of America, 118, e2023301118. 33622786 10.1073/pnas.2023301118 PMC7936330

[CIT0015] De Wardener, H. (1999) Salt reduction and cardiovascular risk: the anatomy of a myth. Journal of Human Hypertension, 13, 1–4. 9928744 10.1038/sj.jhh.1000759

[CIT0016] Diaz Ruiz, C. (2023) Disinformation on digital media platforms: a market-shaping approach. New Media & *Society*, 0, 1–24.

[CIT0017] Ecker, U. K. H., Lewandowsky, S., Cook, J., Schmid, P., Fazio, L. K., Brashier, N. et al. (2022) The psychological drivers of misinformation belief and its resistance to correction. Nature Reviews Psychology, 1, 13–29.

[CIT0018] Filippini, T., Malavolti, M., Whelton, P. K., Naska, A., Orsini, N. and Vinceti, M. (2021) Blood pressure effects of sodium reduction: dose–response meta-analysis of experimental studies. Circulation, 143, 1542–1567. 33586450 10.1161/CIRCULATIONAHA.120.050371 PMC8055199

[CIT0019] Fiocco, A. J., Shatenstein, B., Ferland, G., Payette, H., Belleville, S., Kergoat, M.-J. et al. (2012) Sodium intake and physical activity impact cognitive maintenance in older adults: the NuAge Study. Neurobiology of Aging, 33, 829.e21–829.e28. 10.1016/j.neurobiolaging.2011.07.004 21855174

[CIT0020] Gardener, H., Rundek, T., Wright, C. B., Elkind, M. S. and Sacco, R. L. (2012) Dietary sodium and risk of stroke in the Northern Manhattan study. Stroke, 43, 1200–1205. 22499576 10.1161/STROKEAHA.111.641043 PMC3347890

[CIT0021] Garg, N., Venkatraman, A., Pandey, A. and Kumar, N. (2015) YouTube as a source of information on dialysis: a content analysis. Nephrology (Carlton, Vic.), 20, 315–320. 25641264 10.1111/nep.12397

[CIT0022] Germani, F., Pattison, A. B. and Reinfelde, M. (2022) WHO and digital agencies: how to effectively tackle COVID-19 misinformation online. BMJ Global Health, 7, e009483. 10.1136/bmjgh-2022-009483 PMC9350739 35918074

[CIT0023] Gilmore, A. B., Fabbri, A., Baum, F., Bertscher, A., Bondy, K., Chang, H.-J. et al. (2023) Defining and conceptualising the commercial determinants of health. Lancet (London, England), 401, 1194–1213. 36966782 10.1016/S0140-6736(23)00013-2

[CIT0024] Gwet, K. L. (2008) Computing inter-rater reliability and its variance in the presence of high agreement. The British Journal of Mathematical and Statistical Psychology, 61, 29–48. 18482474 10.1348/000711006X126600

[CIT0025] Hand, R. K., Kenne, D., Wolfram, T. M., Abram, J. K. and Fleming, M. (2016) Assessing the viability of social media for disseminating evidence-based nutrition practice guideline through content analysis of Twitter messages and health professional interviews: an observational study. Journal of Medical Internet Research, 18, e295. 27847349 10.2196/jmir.5811 PMC5128725

[CIT0026] He, J. and Whelton, P. K. (2002) Commentary: salt intake, hypertension and risk of cardiovascular disease: an important public health challenge. International Journal of Epidemiology, 31, 327–331; discussion 331. 11980791

[CIT0027] Hinyard, L. J. and Kreuter, M. W. (2007) Using narrative communication as a tool for health behavior change: a conceptual, theoretical, and empirical overview. Health Education & Behavior, 34, 777–792. 17200094 10.1177/1090198106291963

[CIT0028] Hirawa, N., Umemura, S. and Ito, S. (2019) Viewpoint on guidelines for treatment of hypertension in Japan. Circulation Research, 124, 981–983. 30920921 10.1161/CIRCRESAHA.119.314991

[CIT0029] Holden, C. and Lee, K. (2009) Corporate power and social policy: the political economy of the transnational tobacco companies. Global Social Policy, 9, 328–354. 20228951 10.1177/1468018109343638 PMC2836532

[CIT0030] Hussain, T., Ahmedna, T., Marklund, M., Appel, L. J. and Henry, M. E. (2022) Quality assessment of consumer‐facing websites on sodium reduction. The Journal of Clinical Hypertension, 24, 1285–1292. 36172888 10.1111/jch.14572 PMC9581090

[CIT0031] Jamison, A. M., Broniatowski, D. A., Dredze, M., Wood-Doughty, Z., Khan, D. and Quinn, S. C. (2020) Vaccine-related advertising in the Facebook Ad Archive. Vaccine, 38, 512–520. 31732327 10.1016/j.vaccine.2019.10.066 PMC6954281

[CIT0032] Jernigan, D. H. (2012) Global alcohol producers, science, and policy: the case of the International Center for Alcohol Policies. American Journal of Public Health, 102, 80–89. 22095330 10.2105/AJPH.2011.300269 PMC3490544

[CIT0033] Kendig, M. D. and Morris, M. J. (2019) Reviewing the effects of dietary salt on cognition: mechanisms and future directions. Asia Pacific Journal of Clinical Nutrition, 28, 6–14. 30896408 10.6133/apjcn.201903_28(1).0002

[CIT0034] Kondracki, N. L., Wellman, N. S. and Amundson, D. R. (2002) Content analysis: review of methods and their applications in nutrition education. Journal of Nutrition Education and Behavior, 34, 224–230. 12217266 10.1016/s1499-4046(06)60097-3

[CIT0035] Kumar, N., Pandey, A., Venkatraman, A. and Garg, N. (2014) Are video sharing web sites a useful source of information on hypertension? Journal of the American Society of Hypertension, 8, 481–490. 25064770 10.1016/j.jash.2014.05.001

[CIT0036] Lesser, L. I., Ebbeling, C. B., Goozner, M., Wypij, D. and Ludwig, D. S. (2007) Relationship between funding source and conclusion among nutrition-related scientific articles. PLoS Medicine, 4, e5. 17214504 10.1371/journal.pmed.0040005 PMC1764435

[CIT0037] Lewandowsky, S., Cook, J., Ecker, U. K. H., Albarracín, D., Amazeen, M. A., Kendeou, P., et al. (2020) *The* Debunking Handbook 2020. Libraries at University of Nebraska-Lincoln.

[CIT0038] Lewis, T. (2018) Digital food: from paddock to platform. Communication Research and Practice, 4, 212–228.

[CIT0039] Loomba, S., De Figueiredo, A., Piatek, S. J., De Graaf, K. and Larson, H. J. (2021) Measuring the impact of COVID-19 vaccine misinformation on vaccination intent in the UK and USA. Nature Human Behaviour, 5, 337–348. 10.1038/s41562-021-01056-1 33547453

[CIT0040] Maani, N., Van Schalkwyk, M. C. I., Filippidis, F. T., Knai, C. and Petticrew, M. (2022) Manufacturing doubt: assessing the effects of independent vs industry-sponsored messaging about the harms of fossil fuels, smoking, alcohol, and sugar sweetened beverages. SSM Population Health, 17, 101009. 35036514 10.1016/j.ssmph.2021.101009 PMC8749266

[CIT0041] Mills, K. T., Stefanescu, A. and He, J. (2020) The global epidemiology of hypertension. Nature Reviews Nephrology, 16, 223–237. 32024986 10.1038/s41581-019-0244-2 PMC7998524

[CIT0042] Ministry of Education, Culture, Sports, Science and Technology. (2020) Standard Tables of Food Composition in Japan. https://www.mext.go.jp/a_menu/syokuhinseibun/mext_01110.html (last accessed 8 August 2023).

[CIT0043] Ministry of Health, Labour and Welfare (MHLW). (2019a) Reports of National Health and Nutrition Survey. https://www.mhlw.go.jp/content/10900000/000687163.pdf (last accessed 30 January 2023).

[CIT0044] Ministry of Health, Labour and Welfare (MHLW). (2019b) Report of the Study Group on the Formulation of the ‘Dietary Reference Intakes for Japan People’. 2020. https://www.mhlw.go.jp/stf/newpage_08517.html (last accessed 2 May 2024).

[CIT0045] Ministry of Health, Labour and Welfare (MHLW). (2023) The Third-Term Health Japan 21 Initiative. https://www.mhlw.go.jp/content/001102474.pdf (last accessed 8 July 2023).

[CIT0046] Ministry of Health, Labour and Welfare and the Ministry of Agriculture. (2005) Report of the Food Guide Study Group: Food Guide Spinning Top. Food Guide Study Group. https://www.mhlw.go.jp/bunya/kenkou/eiyou-syokuji.html (last accessed 27 November 2023).

[CIT0047] Ministry of Internal Affairs and Communications, Japan. (2020) Survey Report on Usage Time and Information Behavior of Information and Communication Media. https://www.soumu.go.jp/main_content/000708015.pdf (last accessed 4 March 2023).

[CIT0048] Ministry of Internal Affairs and Communications, Japan. (2021) White Paper on Information and Communications in Japan. https://www.soumu.go.jp/johotsusintokei/whitepaper/ja/r03/pdf/01honpen.pdf (last accessed 26 March 2024).

[CIT0049] Mitsutake, S., Takahashi, Y., Otsuki, A., Umezawa, J., Yaguchi-Saito, A., Saito, J. et al; INFORM Study Group. (2023) Chronic diseases and sociodemographic characteristics associated with online health information seeking and using social networking sites: nationally representative cross-sectional survey in Japan. Journal of Medical Internet Research, 25, e44741. 36862482 10.2196/44741 PMC10020913

[CIT0050] Moodie, R., Stuckler, D., Monteiro, C., Sheron, N., Neal, B., Thamarangsi, T. et al; Lancet NCD Action Group. (2013) Profits and pandemics: prevention of harmful effects of tobacco, alcohol, and ultra-processed food and drink industries. Lancet (London, England), 381, 670–679. 23410611 10.1016/S0140-6736(12)62089-3

[CIT0051] Muggli, M., Hurt, R. and Blanke, D. D. (2003) Science for hire: a tobacco industry strategy to influence public opinion on secondhand smoke. Nicotine & Tobacco Research, 5, 303–314. 12791525 10.1080/1462220031000094169

[CIT0052] Muntzel, M. and Drueke, T. (1992) A comprehensive review of the salt and blood pressure relationship. American Journal of Hypertension, 5, 1S–42S. 1599633 10.1093/ajh/5.4s.1s

[CIT0053] Murakami, K., Shinozaki, N., Kimoto, N., Onodera, H., Oono, F., McCaffrey, T. A. et al. (2023) Web-based content on diet and nutrition written in Japanese: infodemiology study based on Google Trends and Google Search. JMIR Formative Research, 7, e47101. 37971794 10.2196/47101 PMC10690527

[CIT0054] Nadarevic, L., Reber, R., Helmecke, A. J. and Köse, D. (2020) Perceived truth of statements and simulated social media postings: an experimental investigation of source credibility, repeated exposure, and presentation format. Cognitive Research: Principles and Implications, 5, 56. 33175284 10.1186/s41235-020-00251-4 PMC7656226

[CIT0055] Nan, X., Wang, Y. and Thier, K. (2022) Why do people believe health misinformation and who is at risk? A systematic review of individual differences in susceptibility to health misinformation. Social Science & Medicine (1982), 314, 115398. 36327631 10.1016/j.socscimed.2022.115398

[CIT0056] Niino, Y., Mishimura, H., Koga, A., Nakayama, Y. and Haga, M. (2003) Quality of common salt (part II). Journal of Cookery Science of Japan, 36, 107–122.

[CIT0057] Nishiura, H. (2010) A robust statistic AC1 for assessing inter-observer agreement in reliability studies. Japanese Journal of Radiological Technology, 66, 1485–1491. 21099180 10.6009/jjrt.66.1485

[CIT0058] Nomura, S., Sakamoto, H., Ghaznavi, C. and Inoue, M. (2022) Toward a third term of Health Japan 21—implications from the rise in non-communicable disease burden and highly preventable risk factors. The Lancet Regional Health—Western Pacific, 21, 100377. 35098183 10.1016/j.lanwpc.2021.100377 PMC8783949

[CIT0059] Riffe, D., Lacy, S. and Fico, F. (2005) Analyzing Media Messages: Using Quantitative Content Analysis in Research, 2nd edition. Lawrence Erlbaum (LEA communication series), Mahwah, NJ.

[CIT0060] Sasaki, S. (2023) Health and nutrition information and eating behavior. In Kagawa, A. (ed), What is Behavioral Nutrition? Chapter 7. Kagawa Nutrition University, Tokyo, Japan, pp. 284–293.

[CIT0061] Saura, J. R., Reyes-Menendez, A. and Thomas, S. B. (2020) Gaining a deeper understanding of nutrition using social networks and user-generated content. Internet Interventions, 20, 100312. 32300536 10.1016/j.invent.2020.100312 PMC7153295

[CIT0062] Social Insights. (2024). https://sns.userlocal.jp/ (last accessed 4 March 2024).

[CIT0063] Suarez-Lledo, V. and Alvarez-Galvez, J. (2021) Prevalence of health misinformation on social media: systematic review. Journal of Medical Internet Research, 23, e17187. 33470931 10.2196/17187 PMC7857950

[CIT0064] Terada, M., Okuhara, T., Yokota, R., Kiuchi, T. and Murakami, K. (2024) Nutrients and foods recommended for blood pressure control on Twitter in Japan: a content analysis. Journal of Medical Internet Research. doi:10.2196/4907738901016

[CIT0065] Tsuchihashi, T. (2022) Dietary salt intake in Japan—past, present, and future. Hypertension Research, 45, 748–757. 35296804 10.1038/s41440-022-00888-2

[CIT0066] Walter, N. and Tukachinsky, R. (2020) A meta-analytic examination of the continued influence of misinformation in the face of correction: how powerful is it, why does it happen, and how to stop it? Communication Research, 47, 155–177.

[CIT0067] Wang, Y., McKee, M., Torbica, A. and Stuckler, D. (2019) Systematic literature review on the spread of health-related misinformation on social media. Social Science & Medicine (1982), 240, 112552. 31561111 10.1016/j.socscimed.2019.112552 PMC7117034

[CIT0068] Wojcicki, S. (2021) Letter from Susan: Our 2021 Priorities. https://blog.youtube/inside-youtube/letter-from-susan-our-2021-priorities/ (last accessed 5 April 2024).

[CIT0069] Wongpakaran, N., Wongpakaran, T., Wedding, D. and Gwet, K. L. (2013) A comparison of Cohen’s kappa and Gwet’s AC1 when calculating inter-rater reliability coefficients: a study conducted with personality disorder samples. BMC Medical Research Methodology, 13, 61. 23627889 10.1186/1471-2288-13-61 PMC3643869

[CIT0070] World Health Organization (WHO). (2012) Guideline: Sodium Intake for Adults and Children. World Health Organization, Geneva, Switzerland. 23658998

[CIT0071] World Health Organization (WHO). (2020) Salt Reduction. https://www.who.int/news-room/fact-sheets/detail/salt-reduction (last accessed 27 June 2023).

[CIT0072] Zenone, M., Kenworthy, N. and Maani, N. (2022) The social media industry as a commercial determinant of health. International Journal of Health Policy and Management, 1, 1–4. 10.34172/ijhpm.2022.6840 PMC10125226 35490262

[CIT0073] Zhou, B., Carrillo-Larco, R. M., Danaei, G., Riley, L. M., Paciorek, C. J., Stevens, G. A. et al. (2021) Worldwide trends in hypertension prevalence and progress in treatment and control from 1990 to 2019: a pooled analysis of 1201 population-representative studies with 104 million participants. The Lancet, 398, 957–980. 10.1016/S0140-6736(21)01330-1 PMC8446938 34450083

[CIT0074] Zhou, B., Danaei, G., Stevens, G. A., Bixby, H., Taddei, C., Carrillo-Larco, R. M. et al. (2019) Long-term and recent trends in hypertension awareness, treatment, and control in 12 high-income countries: an analysis of 123 nationally representative surveys. The Lancet, 394, 639–651. 10.1016/S0140-6736(19)31145-6 PMC6717084 31327564

[CIT0075] Zhou, L. and Zhang, D. (2007) An ontology-supported misinformation model: toward a digital misinformation library. IEEE Transactions on Systems, Man, and Cybernetics Part A: Systems and Humans, 37, 804–813.

