# Supplementary material for: Misinformation surrounding sodium reduction for blood pressure: content analysis of Japanese posts on X
Source: Health Promot Int. 2024 Jun 27;39(3):daae073. doi: 10.1093/heapro/daae073 (PMC11208930; doi:10.1093/heapro/daae073)
Supplement: daae073_suppl_Supplementary_Material [file daae073_suppl_supplementary_material.docx]

Appendix A. The Keywords We Selected for Searching Posts Related to Hypertension, Nutrients, and Food Consumption.

| Final Keywords | 「(血圧 OR 降圧) (食 OR 栄養 OR 摂 OR 飲 OR 料理 OR 飯 OR ごはん OR サプリ OR メニュー OR 塩 OR アルコール OR 酒)」 | | |
| --- | --- | --- | --- |
| Keywords | Translation | Keywords | Translation |
| 血圧* | blood pressure | 降圧 | lowering blood pressure |
| 食 | food | 栄養 | nutrient |
| 摂** | intake | 飲 | drink |
| 料理 | cooking | 飯 | meal |
| ごはん | diet | サプリ*** | supplement |
| メニュー | menu | 塩 | salt |
| アルコール | alcohol | 酒 | *sake***** |

*Due to the nature of X data, Japanese terms of “血圧” can extract all keywords of high blood pressure, hypertension, lowering blood pressure and blood pressure.

** Due to the nature of X data, Japanese terms of “摂” can extract the word of intake including a Japanese word such as “摂取” and “摂る”.

*** Due to the nature of X data, Japanese terms of “サプリ” can extract the word of intake including both Japanese word of “サプリメント” and “サプリ”.

****In Japanese, there are another term of “sake” for expressing alcohol.

| Appendix B. Themes, Keywords and Post Examples of Misinformation Regarding Sodium Reduction and Blood Pressure Posted in 2022. | | | |
| --- | --- | --- | --- |
| Themes of misinformation | | Examples of Keywords and Codes | Post examples |
|  | Natural Salt | Natural Salt, Sea Salt, Rock Salt, Manufactured Salt, Refined Salt | “Good Salt Doesn’t Raise Blood Pressure: You often hear about reducing salt intake, but this refers to ‘table salt.’ Table salt is mostly composed of sodium chloride. However, high-quality sea salt contains abundant minerals such as magnesium, potassium, and calcium. Consequently, some individuals may actually experience a decrease in blood pressure.” |
|  | Reducing salt could be bad for my health/Salt intake is good for health | Reduced salt intake is bad for health, Salt intake is beneficial for health | “Many people believe that consuming salt raises blood pressure, but it’s actually the opposite. Not consuming enough salt can lead to an increase in blood pressure. A deficiency in salt can result in the inability to deliver blood to the capillaries, which bad for health” |
|  | Salt intake is good for blood pressure | Increased salt intake is good for blood pressure | “Eating natural salt temporarily raises blood pressure, but it eventually returns to normal. Consuming refined salt leads to an initial increase in blood pressure that persists. Avoiding salt causes a temporary drop in blood pressure, but if continued, it eventually rises. Humans originally emerged from the sea, where salt is crucial.” |
|  | Conspiracy theory | The benefit of the Ministry of Health, Labour and Welfare and pharmaceutical companies, Conspiracy | “Encouraging salt reduction weakens the physical and mental well-being of the Japanese population, and the prescription of antihypertensive drugs is motivated by profit. Salt intake and high blood pressure are unrelated, and high blood pressure has little correlation with brain hemorrhage. In that case, what’s the purpose of reducing salt intake and taking antihypertensive medication?” |
|  | Negation of blood pressure medication | Antihypertensive medication, Medication to lower blood pressure | “Reducing salt intake and using antihypertensive medication can lead to an increase in strokes. Antihypertensive drugs are fake.” |
|  | Japanese populations with higher salt intake have longer lifespans | Japanese people, Japanese population live longer | “Do you know why Japanese people who consume a lot of salt seem to live the longest? Reducing salt intake does not only fail to lower blood pressure and prevent strokes, but it also doesn’t have any positive impact on any kind of disease. Feel free to enjoy salt as much as you like.” |
|  | Misconception of blood pressure standards | Blood pressure standard | “Reduce salt intake” is a misconception → Let’s consume mineral-rich natural salt. “High blood pressure” is often a fallacy →A blood pressure of age + 90 is usually considered normal. |
|  | Hot climates or when we exercise | Hot climates, Sweat, Exercise | “We all need salt, especially on hot days or when we sweat a lot. Let’s consume salt as much as we want.” |
|  | Salt Sensitivity | Salt Sensitivity | “Only people with ‘salt sensitivity’ should reduce salt intake.” |
|  | Lack of evidence | No study, No Research, No Evidence | “A meta-analysis showed no relationship between salt intake and high blood pressure” |
|  | Reducing salt intake leads to sodium deficiency | Sodium Deficiency, Lack of Sodium | “Salt serves as a source of minerals. Reducing salt intake can lead to a mineral deficiency. Typically, natural salt does not require significant reduction in consumption.” |
|  | Our body needs sodium | Our body needs sodium, Essential | “Reducing salt intake is challenging because natural salt is essential for humans.” |
|  | Misconception of salt intake standards | Amount of Salt Intake | “The Japan Hypertension Society strongly recommends reducing salt intake to less than 6g per day. However, a worldwide study of 100,000 individuals across 17 countries suggests that a daily salt intake of 10-15g is associated with the lowest mortality rate. This is only half of the recommended amount!” |
|  | No causal relationship with stroke or myocardial infarction | Stroke, Myocardial, Heart Attack | “There is no evidence to suggest that reducing salt intake can decrease the risk of a stroke or heart attack, regardless of whether one has high blood pressure.” |
| Characteristics of Posts | |  |  |
|  | Information sources with citations | Information Sources with Specific Citation | Research says that frequently adding salt to food is linked to a higher risk of premature mortality and reduced life expectancy ([https://dime.jp/genre/1431197/]). Despite this, common advice recommends increased salt intake during hot weather. This creates a contradiction, questioning the necessity of reducing salt intake for everyone. |
|  | Information sources without citations | Information Sources without Specific Citation | “Research shows a link between salt reduction and cognitive decline.” (No additional details about the research are provided in the post.) |
|  | Narrative | Narrative Story | “This is a record of my personal experiment on my own body. For a year, I consumed 10g of salt daily, and on the day of my health check, I drank an additional 10g of salt before getting my blood pressure measured. Surprisingly, my blood pressure had decreased, and my skin symptoms had also improved.” |
|  | Advertisement | Promotion of Specific Products and goods | “It seems that physicians who still recommend ‘salt reduction’ may not understand the difference between table salt and natural salt. Mineral-rich salts like ‘Nuchimasu’ or ‘Shinpo’ salt in your diet can contribute to vitality.” |

| Appendix C. Details of Diseases Mentioned in Posts within the "Reducing salt intake is bad for our health/Salt intake is good for health " theme, n (%). | | | |  |
| --- | --- | --- | --- | --- |
|  | Diseases | n | % |  |
| Reducing salt intake is bad for our health | | 152 | 100.0% |  |
|  | General health | 19 | 12.5% |  |
|  | Dementia | 62 | 40.8% |  |
|  | Cerebral infarction | 51 | 33.6% |  |
|  | Blood circulation | 23 | 15.1% |  |
|  | Skin | 22 | 14.5% |  |
|  | Body temperature | 13 | 8.6% |  |
|  | Constipation | 12 | 7.9% |  |
|  | Kidney | 9 | 5.9% |  |
|  | Immune suppression | 7 | 4.6% |  |
|  | Depression | 6 | 3.9% |  |
|  | Body weights | 6 | 3.9% |  |
|  | Hay fever | 6 | 3.9% |  |
|  | Cancer | 5 | 3.3% |  |
|  | Urinary incontinence | 3 | 2.0% |  |
|  | Myocardial infarction | 3 | 2.0% |  |
|  | Metabolism | 2 | 1.3% |  |
|  | Cerebral hypoxia | 1 | 0.7% |  |
|  | Infertility | 1 | 0.7% |  |
| One post can contain multiple diseases, leading to a cumulative number of posts that may surpass the total count for the theme (n=152). | | | |  |
|  |  |  |  |  |

| Appendix D. Matrix of Misinformation Themes Presented by WHO, AHA, Previous Studies, not Observed in This Study (Continued from Table 2) | | | | | | | |
| --- | --- | --- | --- | --- | --- | --- | --- |
| Themes | Our study | AHA | WHO | Cappuccio FP, 2022 | Cappuccio FP, 2015 | Cappuccio FP, 2014 | Nilay Kumar, 2014 |
| My blood pressure is normal, so I don’t need to worry about how much sodium I eat/Only people with hypertension need to reduce their sodium intake |  | レ |  | レ | レ |  |  |
| Only old people need to worry about how much salt they eat/Only old people need to worry about how much sodium they eat |  |  | レ | レ | レ |  |  |
| Salt added during cooking is not the main source of salt intake. |  |  | レ |  |  |  |  |
| Food does not need salt to have appealing flavor. |  |  | レ |  |  |  |  |
| Food has no flavor without salt/Lower sodium foods have no taste. |  | レ | レ |  |  |  |  |
| Foods high in salt taste salty. |  |  | レ |  |  |  |  |
| I usually don’t salt my food, so I don't eat too much sodium. |  | レ |  |  |  |  |  |
| High levels of sodium are only found in food. |  | レ |  |  |  |  |  |
| I don’t eat a lot of salty food, so I don't eat too much sodium. |  | レ |  |  |  |  |  |
| Sustained reduction in sodium intake is not feasible in free-living individuals |  |  |  | レ |  |  |  |
| Eliminate sodium completely for good health. |  | レ |  |  |  |  |  |
| A reduction in sodium intake below 3.0 g per day activates the renin-angiotensin system |  |  |  | レ | レ | レ |  |
| Consumer taste preferences make change impossible |  |  |  | レ | レ |  |  |
| Food technology cannot change |  |  |  | レ | レ |  |  |
| Food Safety requires the use of salt/Food Safety prevents change |  |  |  | レ | レ |  |  |
| Salt intake in the US has not changed during the past 50 years |  |  |  |  | レ | レ |  |
| Public policy cannot modify salt intake |  |  |  |  | レ | レ |  |
| Traditional highly salted foods do not seem to harm the Portuguese |  |  |  |  | レ |  |  |
| Profits take priority over public health |  |  |  |  | レ |  |  |

| Appendix E. Correlation matrix of Cramer's V coefficients for misinformation themes and characteristics. | | | | | | | | | | | |
| --- | --- | --- | --- | --- | --- | --- | --- | --- | --- | --- | --- |
|  | Natural Salt | Reducing salt could be bad for my health | Salt intake is good for BP | Conspiracy theory | Negation of BP medication | Japanese populations | Misconception of BP | Information sources with citations | Information sources without appropriate citations | Narrative | Advertisement |
| Natural Salt | 1.00 | 0.17 | 0.23 | 0.09 | 0.23 | 0.25 | 0.01 | 0.06 | 0.37 | 0.20 | 0.33 |
| Reducing salt could be bad for my health | 0.17 | 1.00 | 0.02 | 0.00 | 0.43 | 0.23 | 0.03 | 0.05 | 0.14 | 0.03 | 0.04 |
| Salt intake is good for BP | 0.23 | 0.02 | 1.00 | 0.11 | 0.20 | 0.19 | 0.08 | 0.05 | 0.30 | 0.38 | 0.28 |
| Conspiracy theory | 0.09 | 0.00 | 0.11 | 1.00 | 0.12 | 0.16 | 0.11 | 0.04 | 0.21 | 0.15 | 0.11 |
| Negation of BP medication | 0.23 | 0.43 | 0.20 | 0.12 | 1.00 | 0.17 | 0.00 | 0.04 | 0.25 | 0.07 | 0.11 |
| Japanese populations | 0.25 | 0.23 | 0.19 | 0.16 | 0.17 | 1.00 | 0.08 | 0.03 | 0.59 | 0.13 | 0.09 |
| Misconception of BP | 0.01 | 0.03 | 0.08 | 0.11 | 0.00 | 0.08 | 1.00 | 0.02 | 0.13 | 0.00 | 0.00 |
| Information sources with citations | 0.06 | 0.05 | 0.05 | 0.04 | 0.04 | 0.03 | 0.02 | 1.00 | 0.04 | 0.02 | 0.02 |
| Information sources without appropriate citations | 0.37 | 0.14 | 0.30 | 0.21 | 0.25 | 0.59 | 0.13 | 0.04 | 1.00 | 0.26 | 0.14 |
| Narrative | 0.20 | 0.03 | 0.38 | 0.15 | 0.07 | 0.13 | 0.00 | 0.02 | 0.26 | 1.00 | 0.17 |
| Advertisement | 0.33 | 0.04 | 0.28 | 0.11 | 0.11 | 0.09 | 0.00 | 0.02 | 0.14 | 0.17 | 1.00 |
| The highlighted values are considered moderately strong (≥.3). | | | | | | | | | | | |
| BP stands for blood pressure | | | | | | | | | | | |
